# Social creatures: Model animal systems for studying the neuroendocrine mechanisms of social behaviour

**DOI:** 10.1111/jne.12807

**Published:** 2019-11-28

**Authors:** Kelly J. Robinson, Oliver J. Bosch, Gil Levkowitz, Karl Emanuel Busch, Andrew P. Jarman, Mike Ludwig

**Affiliations:** ^1^ Sea Mammal Research Unit Scottish Oceans Institute University of St Andrews St Andrews UK; ^2^ Department of Behavioural and Molecular Neurobiology University of Regensburg Regensburg Germany; ^3^ Department of Molecular Cell Biology Weizmann Institute of Science Rehovot Israel; ^4^ Centre for Discovery Brain Sciences University of Edinburgh Edinburgh UK; ^5^ Centre for Neuroendocrinology Department of Immunology University of Pretoria Pretoria South Africa

**Keywords:** model animals, neuropeptides, oxytocin, social behaviours

## Abstract

The interaction of animals with conspecifics, termed social behaviour, has a major impact on the survival of many vertebrate species. Neuropeptide hormones modulate the underlying physiology that governs social interactions, and many findings concerning the neuroendocrine mechanisms of social behaviours have been extrapolated from animal models to humans. Neurones expressing neuropeptides show similar distribution patterns within the hypothalamic nucleus, even when evolutionarily distant species are compared. During evolution, hypothalamic neuropeptides and releasing hormones have retained not only their structures, but also their biological functions, including their effects on behaviour. Here, we review the current understanding of the mechanisms of social behaviours in several classes of animals, such as worms, insects and fish, as well as laboratory, wild and domesticated mammals.

## 1 INTRODUCTION

Social behaviour is fundamental to the survival of all vertebrates. At the most basic level, reproductive behaviours allow individuals to find each other, mate and produce offspring. Many species have additionally evolved parental behaviours to nurture their young and behaviours that enable living in groups. Within these broad categories, there is a remarkable diversity of social interactions, including affiliative, aggressive, communicative and co‐operative behaviours.^1^ Despite this variation, the occurrence of all social behaviours ultimately depends on the underlying physiology regulating its expression^2^ and aspects of these systems can be highly conserved in structure and function across different species.^3^ It is essential to define these pathways to better understand how social behaviour is perceived and performed by individuals, as well as to uncover why some behaviours are able to adapt to changing environments or social contexts, 4 whereas others are not.^5^

Neuroendocrine systems play an important role in social behaviour because they can act on both the peripheral and central structures needed for its expression. Neuropeptides acting within a variety of brain regions regulate how signals from conspecifics are interpreted and responded to,^6^ whereas hormones acting throughout the body of an individual ensure that the tissues and organs needed to successfully perform social behaviours such as reproduction are present and functioning.^7^ Neuroendocrine systems and the brain structures they act on to promote social behaviour 8 are well conserved across vertebrate species 3 and, in some cases, are also present in invertebrates.^9^ Despite evidence of certain generalities, neuropeptides that affect social behaviours often function in a species‐specific fashion^10^, ^11^ and the behavioural outcome of neuropeptide signalling in distinct brain areas depends on various parameters including sex, reproductive and physiological (i.e., stress‐relevant) state.^12^, ^13^, ^14^ However, comparative neuroendocrine studies can provide insights that are relevant to a wide range of taxa, regardless of the model animal species used, as long as the species being compared both possess the social behaviours and neuroendocrine features that are under investigation.^15^, ^16^

**Figure 1:**
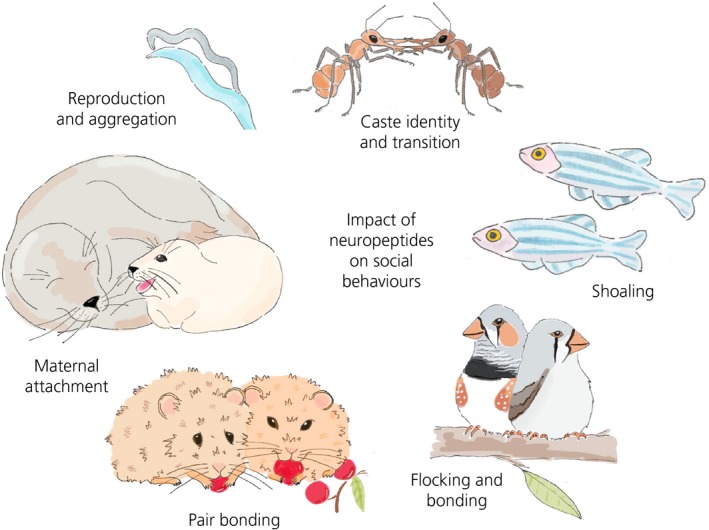
Social behaviours are evolutionarily conserved traits and the neuroendocrine mechanisms underlying them have been studied in many species including those shown here (*Caenorhabditis elegans*,* Harpegnathos saltator*,* Danio rerio*,* Taeniopygia guttata,* *Microtus ochrogaster* and *Halichoerus grypus*)

Model animal species are a key part of neuroendocrine studies and have been used in laboratory settings for decades to investigate the structures and physiological functions underlying social behaviours.^17^ Common animal models include a variety of primate and rodent species 3, various insect and nematode species,^18^ teleost fish, 19 and passerine songbird or quail species (Figure 1).^20^ These laboratory‐bred animal models allow total control of study conditions and minimise potential sources of genetic and experimental variation. However, concern over the lack of ‘real world’ applicability has led to the development of animal models developed from free‐ranging populations in natural environments to validate findings obtained in laboratory studies or to expand our knowledge of social behaviour in relevant ecological contexts.^21^ The use of well‐established laboratory model species and the development of new ecologically relevant animal models will advance our understanding of the neuroendocrine mechanisms regulating social behaviour. Independent of the chosen animal models, three validities should be fulfilled: (i) construct validity (validity of the animal model and of the methods used); (ii) internal validity (quality of the postulated cause‐effect relation); and (iii) external validity (generalisation of the results).^22^ Hence, animal models and their social behaviour need to be stable, reproducible and reliable. Here, we review the species currently used in neuroendocrine research with respect to social behaviours, the insights that these species have provided, and the potential species that could be developed as models in future work.

## 2 NEMATODES

More than four out of five metazoic individuals on earth are considered to be nematodes, and over 25 000 nematode species have been described. Social behaviour in nematodes can take the form of reproductive behaviours such as mating, or group living such as clumping or swarming, and is usually regulated by pheromone signalling.^23^ What we know about how neuropeptides determine nervous system function in nematodes is largely based on studies of *Caenorhabditis elegans*, a free‐living nematode that feeds on bacteria growing on decaying organic matter and populates compost heaps and laboratories worldwide. The nervous system in *C. elegans* hermaphrodites consists of 302 neurones that are stereotypical between individuals and largely have the same connectivity. Although the nervous system is hard‐wired, the behaviour of *C. elegans* must enable these animals to adapt and survive in fluctuating environmental conditions and thus shows a high degree of functional plasticity. Neuropeptide signalling is essential for generating such adaptive behaviour,^24^ and neuropeptides are key regulators and mediators of neural plasticity and learning in diverse behavioural paradigms of *C. elegans*.^25^ In particular, neuropeptide signalling shapes their social and reproductive behaviours.

The best characterised neuropeptide pathway underpinning social behaviour in *C. elegans* hinges on the neuropeptide receptor NPR‐1, a homologue of the neuropeptide Y receptor. In a ground‐breaking study, de Bono and Bargmann 26 showed that NPR‐1 acts as a switch to determine whether animals display social or solitary behaviour during feeding. In the laboratory, worms are maintained on lawns of *Escherichia coli* OP50 bacteria grown on agar dishes. *Caenorhabditis elegans* locate almost entirely on the food lawn and eat most of the time. It was observed that wild *C. elegans* strains prefer to stay at the edge of the bacterial lawn and feed in groups, a behaviour termed ‘aggregation’. This is suppressed in the standard laboratory strain N2, in which animals are solitary and disperse fairly evenly on the lawn. Remarkably, this divergent phenotype depends on a single amino acid difference in NPR‐1 at position 215. The 215V variant in N2 is dominant and represents a gain‐of‐function phenotype in which the receptor has increased activity compared to NPR‐1 in wild strains. The variation has also been implicated in other behavioural differences between N2 and wild strains, such as noxious heat avoidance or ethanol tolerance 27, 28 and the gene thus appears to be a master regulator of behavioural state in *C. elegans*.^29^, ^30^

A series of studies have shown that *npr‐1*‐modulated aggregation behaviour, although it only occurs on food, is not primarily a feeding strategy but, instead, the consequence of a strategy to avoid high ambient oxygen. Several sensory neurones are tonically activated by high ambient [O_2_] and strongly promote increased locomotory speed for as long as the stimulus is presented.^31^ When the *npr‐1^215V^* variant is expressed in specific interneurones (RMG) connected to the O_2_‐sensing neurones, it becomes activated in the presence of food and blocks output, rendering the animals unable to escape oxygen‐rich environments.^31^, ^32^, ^33^ Interestingly, strains of another free‐living nematode species, *Pristionchus pacificus,* which is only distantly related to *C. elegans*, also display oxygen‐induced social behaviour. However, this behaviour is not regulated by *npr‐1*.^34^

The RMG interneurones form the hub of a gap junction network that also connects them with a number of other sensory neurones implicated in aggregation behaviour, including pheromone sensors.^32^ The combination of NPR‐1 activity in RMG and sex also regulates the valence of how other sensory neurones in this circuit control responses to pheromones, by changing the balance between the avoidance‐promoting ADL neurones and the attraction‐promoting ASK neurones.^35^ The RMG/*npr‐1* network is thus a fascinating example of how neuropeptides can integrate information across sensory modalities to regulate social behaviour.

Signalling by the conserved neuropeptide PDF‐1 (pigment dispersing factor) in the *C. elegans* nervous system plays key roles in modulating sexually dimorphic behaviours related to reproduction. Its receptor, PDFR‐1, is orthologous to the secretin family of G‐protein‐coupled neuropeptide receptors. PDF‐1 regulates decision‐making specifically in males: well‐fed male *C. elegans* frequently leave a source of plentiful food when hermaphrodites are absent, in search of a mating partner, whereas hermaphrodites show little tendency to leave food under the same conditions. This male‐specific mate‐searching behaviour requires PDFR‐1 receptor expression in specific sensory neurones.^36^ It thus appears that the PDF‐1 pathway regulates the sexually dimorphic motivational state and promotes goal‐oriented exploratory behaviour, by modifying the way sensory input is processed. PDF signalling has also been implicated in regulating the reproductive drive in other invertebrates, such as rival‐induced prolonged male mating in *Drosophila*.^37^

PDF‐1 is also expressed in a recently discovered interneurone pair, MCM, which is found only in males. The MCM neurones are specifically required for male‐specific associative learning: hermaphrodite *C. elegans* learn to avoid NaCl if it is presented to them in the absence of food. Males suppress this avoidance if NaCl is presented in the absence of food but in the presence of hermaphrodite mating partners. This sexual conditioning, over‐riding the effect of starvation on chemosensory learning, is disrupted by MCM ablation and in *pdf‐1* null mutants.^38^

Recent studies have highlighted a remarkable degree of conservation of the oxytocin/vasopressin signalling system in the regulation of nematode behaviours related to reproduction.^6^ Mating in *C. elegans* is conducted primarily by the male, which, when touching a hermaphrodite with his tail, initiates a series of mating behaviours during which he makes turns sliding around her body until he locates the vulva with his tail, stops moving and then transfers sperm.^39^ The *C. elegans ntc‐1* gene encodes the neuropeptide nematocin, which is homologous to mammalian vasopressin and oxytocin. Nematocin signalling has multiple roles in these male‐specific behaviours and is necessary for reproductive efficiency. *ntc‐1* is expressed in thermosensory, mechanosensory and male‐specific CP motoneurones, whereas the receptors are expressed, amongst other cells, in male‐specific neurones and muscles coordinating mating behaviour, including sensory neurones that detect contact with hermaphrodites or the vulva. Mutants of *ntc‐1* are less efficient in mate searching, mate contact, locating the vulva and transferring sperm, frequently repeating individual steps in this behavioural sequence. Overall, nematocin signalling appears to organise the function of distributed circuits to coordinate individual behavioural programmes into coherent reproductive behaviour in male *C. elegans*.^40^

Nematocin signalling is also required for an intriguing social interaction of *C. elegans* with their offspring, in which a pheromone signal emanating from the larvae increases the propensity of adult *C. elegans* to leave the bacterial food. This behaviour is not caused by depletion of the bacteria and is conspecific, with larvae of related nematode species not increasing food‐leaving behaviour in *C. elegans*. It is absent in sterile adults, in mutants unable to produce pheromones and in loss‐of‐function mutants of the *ntc‐1* nematocin. In a reproducing population with increasing density, this form of ‘parental leave’ may increase the fitness of their offspring by making more food available to them; however, this has not been demonstrated directly.^41^

To conclude, studies on neuropeptide function in *C. elegans* have clearly shown that they are of profound importance for the modulation of neural circuit activity in social behaviours, and also that specific neuroendocrine systems have roles in similar tasks across even distantly related metazoa.^42^ In the future, it will be exciting to learn more about the role of neuropeptide‐regulated behaviours from studies of the ecology of *C. elegans* and hopefully other nematodes under more natural conditions. As a result of its highly mapped and invariant nervous system, *C. elegans* could also be useful for better understanding the specificity of neuropeptide signalling, such as where a particular neuropeptide acts relative to its release site.

## 3 INSECTS

Insects are rich in social behaviours. For many insects, this is most apparent in elaborate courtship behaviours leading to mating; in others, parental care can take a variety of forms. At the pinnacle are the variety of social interactions that underpin the structure and function of social insect colonies (bees, wasps, ants and termites). Neuropeptides are said to be the largest single class of signal molecule in insects,^43^ with a variety of roles in metabolism, development, homeostasis and behaviour; a review of neuropeptides and behaviour is provided elsewhere.^43^ Over 150 insect neuropeptides have been identified. To some extent, linking neuropeptides to the control and modulation of insect social behaviours is a field that is still in its infancy. Nevertheless, some striking advances have been made.

Although not the most social of insects, even the laboratory fruit fly *Drosophila melanogaster* has sophisticated social behaviours connected to its courtship and mating ritual. This has been subjected to intense analysis that has uncovered roles for neuropeptides. Sex peptide is produced in the male accessory gland and, during copulation, it is transferred in the seminal fluid to the female, where it induces egg laying behaviour and loss of receptivity to additional courting males.^44^, ^45^ Males also leave behind an anti‐aphrodisiac pheromone on mated females that deters other males.^46^ Triggering the response of the deterred male requires tachykinin (TK) in its brain. Interestingly, activation of the TK gene in male *Drosophila* also increased male‐male aggression when competing for mates. When TK+ neurones were activated in the male brain, this resulted in increased aggression in the presence of males and courtship in the presence of females.^47^ This convergence of aggression and sex in a common pathway suggests that TK release (triggered partly by pheromones) modulates the choice between fight or courtship.^43^

Although insects often have a hands‐off approach to raising their offspring (perhaps limited to depositing eggs in a conducive location: ‘shoot and scoot’), many insects exhibit a variety of sophisticated methods of parental care, often entailing specific social behaviours.^48^ This is a promising area for neuropeptide research. The burying beetle *Nicrophorus vespilloides* has the interesting behaviour of feeding pre‐digested carrion to its larval offspring when they solicit it. This may be considered as requiring an inhibition and reversal of the parent's normal motivation to eat. In *Drosophila* and other insects, neuropeptide F (related to vertebrate neuropeptide Y) has been associated with foraging and feeding behaviours,^43^, ^49^, ^50^, ^51^ and thus is a candidate for regulating the parental behaviour of *Nicrophorus*. Indeed, Cunningham et al^52^ showed that adult expression of the neuropeptide F receptor is greatly reduced during parental care.

Social insects (bees, wasps, ants and termites) are important emerging models for understanding the genetic regulation of social behaviour. Interestingly, in comparisons of neuropeptides (computationally predicted and biochemically confirmed), it appears that honeybees may express fewer neuropeptide forms than do basal solitary insects.^53^ Thus, the increased sophistication of social behaviours derives from an expansion of functions for existing neuropeptides rather than an expansion of neuropeptide repertoires. Nevertheless, the social structures of insect colonies are potentially rich sources for understanding the role of neuropeptides in social behaviours.^43^

Honeybees (*Apis mellifera*) have been well researched with regard to their social structure, from the time of the discovery of their famous ‘waggle dance’ onwards. In several studies, proteomic analyses have been used to identify neuropeptides associated with behaviours. In most cases, expression differences are found, although the causality is not established.^54^, ^55^ A possible exception is control of aggressive behaviour in Africanised honeybees. These so‐called ‘killer bees’ were bred in the 1950s in Brazil by crossing African and European strains of honeybee. Their heightened aggressiveness compared with the parental strains is likely driven by differences in neuropeptide expression. To determine possible neuropeptide involvement, proteomic analyses were conducted to compare neuropeptide expression in brains from aggressive and passive bees.^56^ This identified, in ‘aggressive’ brains, an increased degree of processing of a protein precursor to yield allatostatin A (AST‐A) and tachykinin‐related neuropeptides. When these were injected into young (and therefore passive) bees, they too became aggressive. Interestingly, in other insects AST‐A is linked to increased feeding and foraging behaviours.^43^

At the highest level of insect social behaviours, eusocial insects are characterised by having a division of labour in females between reproductive ‘queen’ and sterile ‘worker’ castes. In the vast majority of species, queens and workers are genetically identical but differ in anatomy and in their behavioural repertoires. For example, queens remain within the protection of the nest, whereas workers are responsible for foraging, defence and brood care. In most species, these differences are determined epigenetically during development. In the ant species *Harpegnathos saltator*, however, even adult workers have the ability to transition to becoming an egg‐layer (a ‘gamergate’), suppressing similar behaviour by fellow workers in the process (including intimidating them by fighting). Comparison of the transcriptomes of brains from gamergates and regular workers identified the neuropeptide corazonin as strongly associated with workers.^57^ Invertebrate corazonin is part of the gonadotrophin‐releasing hormone superfamily. It is present in most insects (aphids and beetles being exceptions) and, amongst other things, has been implicated in stress responses in *Drosophila*.^58^ However, in the case of *Harpegnathos*, it appears that corazonin specifically promotes foraging activity: injection of corazonin into ants that were transitioning to gamergate status strongly promoted worker‐like foraging activity.^57^ High levels of corazonin were also found in the workers of other ants and wasps, showing that this is not a peculiarity of the unusual social structure of *Harpegnathos*.^57^

Social insects represent a vast resource for understanding neuropeptide functions. The genetic dissection of complex social behaviours has thus far been prominent in *Drosophila*. This will undoubtedly be broadened to other insects in the future by the application of techniques such as RNA interference and CRISPR/Cas9 gene editing.

## 4 FISH

Teleost fishes are the most diverse and largest existing vertebrate taxa, with tens of thousands of species described so far, including the majority of fish species targeted by commercial fishing and aquaculture. As such, they display a large variety of social behaviours. For example, different teleost species may employ a variety of feeding and mating strategies, parental care behaviours and social hierarchies. They may differ in their levels of aggression and territoriality and employ a variety of social cues, including visual, olfactory and sound stimuli. As with other species, social behaviour in fish is also dependent on their internal state, especially hormonal levels.^59^, ^60^ Lastly, the ecology of social behaviour in teleost fishes has been extensively reviewed.^61^

Here, we focus on the zebrafish (*Danio rerio*), a small fresh‐water fish of the Cyprinidae family that is extensively studied as a model for neurodevelopment, physiology and animal behaviour because of the ease of accommodating large numbers of fish in a laboratory, as well as the availability of genetic tools and ethological assays.^62^ The zebrafish geographical range has been documented in Pakistan, Myanmar, Nepal and India, and its natural habitat spans rivers and ponds near streams and rice paddies.^63^ Zebrafish are a social species in that they display collective behaviour in the formation of small, loose groups, known as shoals. The benefits of shoaling behaviour have been attributed to predator avoidance, increased success in foraging and mating, and higher locomotion efficiency. The size of shoals in the wild is highly variable and depends on the attributes of the specific body of water (eg, size, amount and type of cover, current speed, etc.). Shoals comprise between four and 12 fish in small, slow flowing creeks and reach up to 300 individuals in fast‐flowing rivers.^64^ Notably, under certain laboratory settings, small groups of zebrafish also exhibit synchronised motion known as ‘schooling’, and it has been suggested that this type of collective swimming is influenced by their environment and the level of stress.^65^

Zebrafish females lay eggs that are externally fertilised and the larvae hatch approximately 3 days after fertilisation. By 5‐6 days, the larvae have a functional endocrine system and exhibit complex behaviours such as prey capture, escape and stress responses.^66^ In a laboratory setting, a preference to swim near conspecifics is observed, starting in three week‐old juveniles^67^; however, the ontogeny of collective behaviours is established earlier because zebrafish larvae already display weak attraction toward each other from 7 days post‐fertilisation and this interaction increases with age.^68^

Over the last decade, several behavioural paradigms in a laboratory setting have been developed. Zebrafish use both visual and olfactory social cues; however, as a result of technical difficulties in controlling the local concentration of water‐soluble odourants, most paradigms to measure zebrafish social‐cognitive abilities rely on visual cues.^62^ They can identify shoal‐mates by their skin colour patterns and this behaviour is influenced by early‐life experience.^69^ Assessment of different social modalities is performed by a variety of social behaviour assays. Thus, the “cohesiveness” of a shoal of fish has been shown to be context‐dependent, increasing in the presence of a predator and diminishing during feeding.^63^, ^70^ The motivation or social drive to swim in a group can be measured by the visually‐mediated social preference test, in which a single fish has the choice of swimming near a shoal compartment containing conspecifics or near an empty “no shoal” compartment.^71^ A higher level of social‐cognitive appraisal relates to discrimination between individual conspecifics. This can be measured by the visually mediated social recognition test, which measures the preference of a focal fish for a novel versus a familiar conspecific.^72^ Recent studies used computer animations to investigate which specific visual features zebrafish use to appraise and react to social cues, such as conspecific form and biological motion.^73^, ^74^, ^75^

Studies of neuroendocrine signals, which modulate zebrafish social drive, memory and perception, are beginning to emerge. The structure and function of the neurohypophyseal hormones oxytocin and vasopressin are evolutionarily conserved among many species.^71^ Zebrafish injected with either oxytocin or vasopressin display increased social preference and reduced predator fear.^76^ An additional level of evolutionary conservation is the genetic determinant of oxytocin neurone development. The neuroendocrine transcription factor, Orthopedia, regulates co‐expression of oxytocin and the stress neurohormone corticotrophin‐releasing hormone.^71^ Moreover, developmental mutations in Orthopedia affect both stress and social behaviours throughout life, suggesting that neuropeptide balance in discrete hypothalamic neurones may have a long‐term effect on adult social preference.^71^

Recent studies have begun to dissect specific neural circuits underlying social behaviour. Dyads of two males display aggressive behaviour to establish hierarchical dominant‐subordinate relationships even in the absence of competition for food, shelter, or a potential mate. The outcome of a single fighting interaction is enough to induce experience‐dependent shifts in social status.^77^ Chou et al^78^ showed that sub‐regions of the dorsal habenula antagonistically regulate the outcome of such social conflict. Because the neuropeptide vasotocin/vasopressin is associated with dominant‐subordinate relationships,^79^ it would be interesting to test whether neurones in the dorsal habenula receive inputs from vasotocin/vasopressin neurones.

The zebrafish is an excellent model for social neuroscience research as it exhibits a variety of measurable social behaviours. As in other animals, these social behaviours are highly dependent on external environmental cues (eg, size of the arena, water flow, etc.), internal state (eg, stress level, hunger state, etc.) and genetics, and the fundamental principles and mechanisms underlying zebrafish social behaviour are evolutionarily conserved. As an animal model that is readily amenable to genetic perturbations, the zebrafish is useful for identifying genes involved in the formation and function of the neuronal circuits that underlie social behaviours. Furthermore, zebrafish are uniquely suitable for detailed, high‐resolution brain activity imaging, as their embryos and larvae develop externally and are optically transparent.^66^ Hence, using the above‐described paradigms in combination with state‐of‐the art optogenetic and imaging tools will allow future understanding of the mechanisms by which the vertebrate brain receives and processes socially‐relevant information.

## 5 BIRDS

Many bird species, such as passerine songbirds and quails, are used to investigate social behaviours, both in the wild and in captivity. Much of our knowledge of the social behaviour network (which consists of basal forebrain and midbrain structures containing a set of interconnected nuclei that control social behaviour) 80 originates from studies on birds, which show seasonal expression of reproductive behaviour, marked sexual behavioural dimorphism and responses to social behaviour (song/displays, etc.) in naturalistic contexts. The core components of the brain's social behaviour network are strikingly similar across vertebrate groups and are essential for the regulation of fundamental behavioural features such as maternal care, sexuality, communication and aggression.^81^ The brain areas originally implicated are the medial amygdala, medial bed nucleus of the stria terminalis, preoptic area, lateral septum, ventromedial and anterior hypothalamus, and the midbrain periaqueductal grey area and tegmentum.^80^ These areas are all mutually connected and use numerous hormones (including in particular prolactin and steroids) and peptides (including gonadotrophin‐releasing hormone, gonadotrophin‐inhibitory hormone, neurotensin, opioids and vasoactive intestinal polypeptide), all of which appear to be relevant to individual, species and seasonal differences in social structure.^82^, ^83^, ^84^, ^85^

Recently, the mesolimbic dopamine system and the paraventricular nucleus have been added to the network. The paraventricular nucleus is an important source of nonapeptide projections and almost all forms of social behaviour regulated by the social behaviour network are modulated by the vasopressin‐ and oxytocin‐like peptides (mesotocin and vasotocin in birds), including parental behaviour, pair bonding, sexual behaviour, social recognition, non‐sexual affiliation and aggression. In zebra finches, social behaviours are not only correlated with activation of neuropeptide receptors in regions of the common social behaviour network, but also involve vocal and auditory circuits 86, 87 and antagonism of the signalling of vasopressin‐like peptides alters vocal learning.^88^

Most bird species and some mammals (see below), including humans, are socially monogamous and exhibit biparental care. The simultaneous evolution of multiple behavioural characteristics is associated with evolutionary convergence in the anatomy of nonapeptide systems and their behavioural effects. For example, mesotocin and oxytocin affect maternal care in mammals and neognathan birds, as well as pair‐bonding in prairie voles and zebra finches. The behavioural and physiological effects of avian nonapeptides are mediated by a suite of four receptor types (VT1‐VT4) that show strong sequence similarities with respect to those of mammals and other vertebrates and, as is typical of mammals, the distributions of these binding sites are highly species‐specific. Much of the behavioural diversity observed is produced by variations in gene expression, rather than by large‐scale reorganisations of social circuitry or major differences in anatomy.

Studies in the socially monogamous zebra finch showed that systemic administration of an oxytocin antagonist significantly reduced the likelihood of pairing in inexperienced birds.^89^, ^90^ Recent work also suggests that the nonapeptides play a role in initial pair formation that is different from that in pair maintenance.^91^ Vasopressin and oxytocin are important mediators of parental behaviour in mammals. In birds, seasonal expression of reproductive behaviour induces a male‐biased dimorphism in the vasotocin circuitry in the brain, specifically in the medial bed nucleus of the stria terminalis. In seasonally breeding birds, the circuit diminishes during non‐breeding periods and its role may lie in reducing male aggression during the breeding season, to stimulate heightened affiliative behaviour.^92^

Activation of nonapeptide receptors by endogenous mesotocin also promotes social behaviour (preferences for larger groups) and the preference for familiar social partners in the gregarious zebra finch.^93^, ^94^, ^95^ Antagonism of oxytocin receptors also reduces the preference for larger groups in finches.^96^ Isotocin modulates social communication and approach in fishes^97^ and mesotocin promotes social behaviour in birds,^93^ suggesting that oxytocin‐like peptides affect social groupings in different vertebrate groups. Grouping behaviour follows seasonal variation in many bird species, with shifts towards territoriality in the breeding season and grouping in the winter. The receptor densities in various brain regions vary seasonally, particularly the densities of receptors for the neuropeptides vasotocin, corticotrophin‐releasing hormone and vasoactive intestinal polypeptide (VIP), and VIP receptor density is associated with seasonal flocking.^95^

A substantial number of other neuropeptides and brain signalling molecules are correlated with nonapeptide actions and social behaviour. For example, studies within socially diverse species of estrildid finches and emberisid sparrows suggest a role for VIP not only in avian grouping behaviour but also in aggression and parental care.^84^

Here, we touch only on the most recent evidence for nonapeptide actions on pair bonds and social behaviour. There is also some evidence for vasotocin and mesotocin involvement in parental behaviour,^92^, ^98^ territorial aggression and competitive aggression for mates.^14^ Because several aspects of the nonapeptide systems are evolutionarily conserved across vertebrate taxa, future discoveries made in birds may guide the development of hypotheses and predictions for subsequent investigations across a much wider array of taxa.

## 6 LABORATORY RODENTS

The classical rodent models in neuroscience are laboratory rats and mice,^3^ which are chosen not only because of their relatively easy breeding and fast reproduction cycles, but also, more importantly, because of their translational relevance, partly based on the depth to which these rodent models have been studied.^99^ These models helped us to deepen our understanding of the neurobiology of social behaviour. Over the past decades, laboratory rodents have been studied in terms of social memory and recognition,^100^ and various forms of social interactions including sexual behaviour,^101^, ^102^ parental care,^6^, ^103^ social play^104^ and offensive/defensive aggression.^101^, ^105^ Especially, the specific roles of neuropeptides in social behaviour have been uncovered by studying rodents. For example, we have learned that high activities of both the oxytocin and vasopressin systems are necessary to initiate and maintain adequate maternal care 106 and maternal aggression against potential threats.^105^ In turn, the brain stress system needs to be dampened, otherwise the mother neglects her offspring.^107^ The latest addition to that list of studied social behaviour is a behavioural model for social fear; a mouse of either sex is conditioned (mild foot shock) against a conspecific, resulting in social avoidance behaviour.^108^ This behaviour is triggered by the brain oxytocin system; its heightened activity is able to buffer against social fear, either by artificially increasing brain oxytocin levels 109 or in a state of high innate oxytocin activity (i.e., during lactation).^110^ However, it is important to remember that the roles of oxytocin and vasopressin in social behaviour are not generally applicable and, instead, are brain site‐specific and sex‐dependent.^111^

Other species that have been used in social behaviour research include mole rats, California and singing mice, and meadow and prairie voles.^103^, ^112^, ^113^, ^114^, ^115^, ^116^ Prairie voles especially became an important animal model for studying pair‐bonds, social support/consoling behaviour and the consequences of social loss and/or biparental care. For example, increased activity of vasopressin and oxytocin facilitates bonding to a partner, whereas sudden disruption of an established pair bond leads to impaired oxytocin signalling as a result of increased activity of the stress system.^112^ Furthermore, when one prairie vole partner is briefly separated and stressed (immobilisation or paired shock/tone), the subsequent reunion with the partner results in increased grooming (consolation behaviour),^117^ which in turn causes a faster recovery from the experienced stress,^118^ and increased oxytocin signalling is the main mediator in both cases.

Several of these behaviours have also been studied in other laboratory mammals, including less prominent animal models, such as the socially monogamous and biparental titi monkey.^119^ Research on the neurobiological basis of their complex social behaviour has become more prominent in recent years as a result of their potential translational importance with resepect to understanding the neural basis of disorders of social behaviours (including autism).^11^

As in other disciplines of neuroscience, social neuroscience has incorporated transgenic approaches, with a growing number of transgenic mouse and rat models, and recently even transgenic prairie voles. Such transgenic models range from full knockout or knock‐in of single or multiple genes to brain region‐ and gene‐specific inhibition or activation as a result of targeted modifications, eg, optogenetics or DREADD (designer receptor exclusively activated by designer drug). Furthermore, the role of epigenetics in social behaviour, as well as the effects of targeted manipulations, has been increasingly studied from the mid‐2000s onwards, providing us with insights into the behavioural effects of stressful or traumatic events, which even persists into the next generations.^120^ Therefore, studying social behaviour and especially its neurobiological basis in rodent animal models profits from the emerging new techniques in the field.

## 7 WILD AND DOMESTIC MAMMALS

The vast majority of research on the neuroendocrinological systems modulating social behaviour occurs in laboratory animal species.^3^ However, there is growing interest in studying these mechanisms outside the laboratory, leading to an increase in studies with domestic or wildlife species. Both domestic and wildlife species show a diverse range of social behaviours in various contexts, including recognition of conspecifics, aggression, living in groups, dominance hierarchies, attracting and courting mates, parental or alloparental care, bonding across species boundaries, and even complex social traits such as altruism. Adapting laboratory methodologies to species in natural contexts presents many challenges. Nonetheless, new animal species for investigating neuroendocrine drivers of social behaviour are validated every year, using species whose natural behaviour allows investigation of particular social phenomena.

Domestic model species have been used to investigate both central and peripheral neuroendocrine systems. This has provided not only insights that are applicable to more conventional mammalian model species, but also evidence that informs the commercial practises for breeding and rearing these animals.^121^ Historically, research on sheep has been particularly valuable for uncovering the central pathways regulating the bonding processes between mothers and infants and the subsequent expression of maternal behaviour.^122^, ^123^ These studies use techniques such as microdialysis and intracerebroventricular infusions to measure and manipulate neuropeptides in various brain regions of breeding ewes, and they have demonstrated the importance of oxytocin in modulating maternal behaviour.^3^) In the last 5 years, there has been a surge of interest in studying social neuropeptides such as oxytocin in companion domestic animals. One study giving intranasal oxytocin and measuring levels in the urine of pet dogs and their human owners provided the first evidence of positive‐feedback loops acting across bonded individuals.^124^ However, some papers contributing to this avenue of research must be viewed with caution, as validation work for some of these species can be incomplete or not full reported, such as omitting to conduct parralism testing to determine matrix effects. 125

There are far fewer studies using wildlife species to document neuroendocrine systems because experiments are limited to measuring peripheral concentrations of neuropeptides in blood, urine or saliva and using peripheral or intranasal manipulations. Despite these limitations, wildlife species can present excellent systems by which to explore the physiology regulating various social behaviours because certain species evolve to be highly reliant on particular social acts such as alloparental care and reciprocal altruism. Oxytocin manipulations have been used in several exotic species to investigate the physiology underlying living in co‐operative groups.^7^ Meerkats (*Suricata suricatta*) and naked mole rats (*Heterocephalus glaber*) both live in groups that support the breeding efforts of a single dominant breeding female. Oxytocin given to meerkats increased co‐operative behaviour and care‐giving to pups,^126^ whereas oxytocin given to naked mole rats increased pro‐social behaviours.^127^ Reciprocal altruism is essential for survival in vampire bats (*Desmodus rotundus*) because it enables the sharing of blood feeds between roost companions, and oxytocin manipulations were reported to increase pro‐social behaviour and food sharing between individuals.^128^

A number of primate studies have also provided evidence for the role of oxytocin with respect to promoting pro‐social behaviour among group members. Intranasal manipulations increased pro‐social behaviours within pair bonded captive marmosets (*Callithrix penicillate*)^129^ and salivary and urinary oxytocin concentrations detected in captive western lowland gorillas (*Gorilla gorilla gorilla*) varied depending on the social context prior to sample collection.^130^ Studies on wild populations of chimpanzees (*Pan troglodytes*) have also successfully detected correlations between oxytocin and pro‐social behaviours, such as grooming,^131^ food sharing 132 and group cohesion prior to intergroup conflict.^133^

A marine mammal model system for investigating oxytocin functionality in natural environments has been developed using wild grey seals (*Halichoerus grypus*). Blood samples collected from mother‐pup pairs showed a positive relationship between plasma oxytocin levels and mother‐pup proximity in a breeding colony.^134^ The causality of this neuropeptide‐behaviour relationship has also been determined by i.v. manipulation experiments in the wild, which showed that oxytocin stimulates proximity seeking and other pro‐social behaviours in seals.^7^ A recent seal study has also provided evidence of positive feedback loops acting across mother‐infant pairs and demonstrated a relationship between plasma oxytocin levels and daily mass gain in pups, without increased energetic expenditure by the mother.^135^ Although marine mammals appear to be an unlikely choice of species for neuroendocrine research, several seal species breed on land and are also individually identifiable and return each year to the same place to give birth, enabling repeated blood sampling alongside observation of the entire pup rearing period.^134^

There are currently few examples of animal species outside of classic laboratory models that have been used to investigate neuroendocrine impacts on social behaviour in natural environments. However, every year, methods of investigating species novel to the field are validated both in captivity (eg, gorillas 130 and wolves [*Canis lupus*] 136) and in the wild (eg, bottlenose dolphin *Tursiops truncates* 137). Wildlife and domestic species can provide unique opportunities to investigate physiological drivers of social behaviour outside of laboratory environments or in completely natural systems and thus future work will broaden the range of species available for such research at the same time as providing insights into neuropeptide functionality that can be applied across more conventional study species and humans.

## 8 CONCLUSIONS

The list of ‘social creatures’ is long, and this review covers only a limited range of current studies that have expanded our basic understanding of the neuroendocrine mechanisms of social behaviour. In recent years, there has been an increase in translational medicine utilising neuropeptide research to identify new strategies and therapeutic interventions for current major psychological conditions, including autism and depression. The ability of neuroendocrine studies to help treat these conditions has raised the importance of determining the normal and pathological mechanisms and pathways underpinning these disorders. At the same time, it is crucial to the success of these treatments to identify species and contexts where neuropeptides have opposing functions in social behaviours, such as the contrasting role of oxytocin with respect to mediating aggressive behaviour across different species, sexes and reproductive or social contexts.^3^ Transgenic animals, including mice, rats, *Drosophila*, *C. elegans* and the zebrafish, are amenable to genetic manipulation and analysis and, together with the use of state‐of‐art techniques (eg, optogenetics and pharmacogenetics), allow us to scrutinise neuroendocrine systems in‐depth, unravelling complex interactions among the neural, hormonal and peripheral systems that underlie physiological functions and social behaviours.^138^ Although some of these model animals are well established and widely used to address numerous questions, wild animal populations of species ranging from invertebrates to large vertebrates are important for the investigation of specific physiological processes and behaviours in their natural environments.^138^
